# Supplementary material for: Surprise-minimization as a solution to the structural credit assignment problem
Source: PLoS Comput Biol. 2024 May 28;20(5):e1012175. doi: 10.1371/journal.pcbi.1012175 (PMC11175464; doi:10.1371/journal.pcbi.1012175)
Supplement: S1 Supporting Information — Table A: Model comparison for the different model variants with surprise minimization. In a separate analysis, we identified the model with the surprise-minimization that best explains the behavioral data. We considered three different mechanisms for updating unchosen options: (1) Learning-rate based decay, (2) free-parameter decay and (3) counterfactual updating. Counterfactual updating is supported by the anti-correlated nature of the random walk procedure for each decision, as well as the model selection procedure (XP = 0.71). Fig A: Model-based regression analysis for reward prediction errors split into valence and surprise components. In a separate model-based EEG analysis we split the (signed) prediction errors in its constituent parts–valence and surprise–for both the correct and incorrect policy. (PDF) [file pcbi.1012175.s001.pdf]

Surprise-minimization as a solution to the structural credit assignment problem

Franz Wurm[1,2,3], Benjamin Ernst[1], & Marco Steinhauser[1]

[1] Catholic University of Eichstätt-Ingolstadt, Germany; [2] Leiden University, the Netherlands;

[3] Leiden Institute for Brain and Cognition, Leiden University, Leiden, the Netherlands

* f.r.wurm@fsw.leidenuniv.nl

Supporting Information:

Supplementary Figures and Tables

Model comparison for the surprise minimization mechanism

In a separate analysis, we identified the model with the surprise-minimization that best explains the behavioral data. The surprise minimization and behavioral arbitration algorithm is identical for all models. We considered three different mechanisms for updating unchosen options: (1) Learning-rate-based decay decays the action values for unchosen options towards zero based on the inverse learning rate (1-α), effectively coupling updating of chosen and unchosen options. (2) Free decay introduces a free learning decay parameter that decays action values for unchosen options towards zero, effectively uncoupling updating of chosen and unchosen options. (3) Counterfactual updating does not decay unchosen options, but updates them based on the prediction errors. While chosen options are updated by adding the weighted prediction error (+ α * PE), unchosen options are updated by subtracting the weighted prediction error (- α * PE). Counterfactual updating effectively makes the action values for each decision symmetrical. Interestingly, counterfactual updating is supported by the anti-correlated nature of the random walk procedure of outcomes, as well as the model selection procedure (Table A in S1 Supporting Information).

Additionally, we have implemented each model with a free starting point for arbitration weights. In contrast to the fixed starting point at 0.5, a free parameter for the arbitration weight allows to capture potential starting biases (e.g., increased attention for one policy). Importantly, model selection clearly favored models without the free starting bias.

In conclusion, surprise minimization with counterfactual updating of unchosen options and a fixed starting point for arbitration weight is favored for AIC, BIC and exceedance probability.

Table A. Model comparison for the different model variants with surprise minimization

			Behavioral study				EEG study			
Decay	Start point	#	-LL	BIC	AIC	xp	-LL	BIC	AIC	xp
(1-α)	0.5	4	14596	30288	29577	0.22	6682	14004	13589	<0.01
(1-α)	free	5	14547	30463	29574	0.01	6643	14085	13566	<0.01
free	0.5	5	14556	30482	29593	<0.01	6658	14116	13597	<0.01
free	free	6	14499	30642	29575	<0.01	6619	14197	13575	<0.01
CF	0.5	4	14561	30218	29507	0.75	6666	13972	13557	0.71
CF	free	5	14510	30390	29501	0.01	6627	14054	13535	0.29

Notes. # is the number of free parameters within a model, α is the learning rate, CF is counterfactual updating, -LL is the negative log likelihood, BIC is the Bayesian Information Criterion, AIC is the Akaike Information Criterion, xp is the exceedance probability.

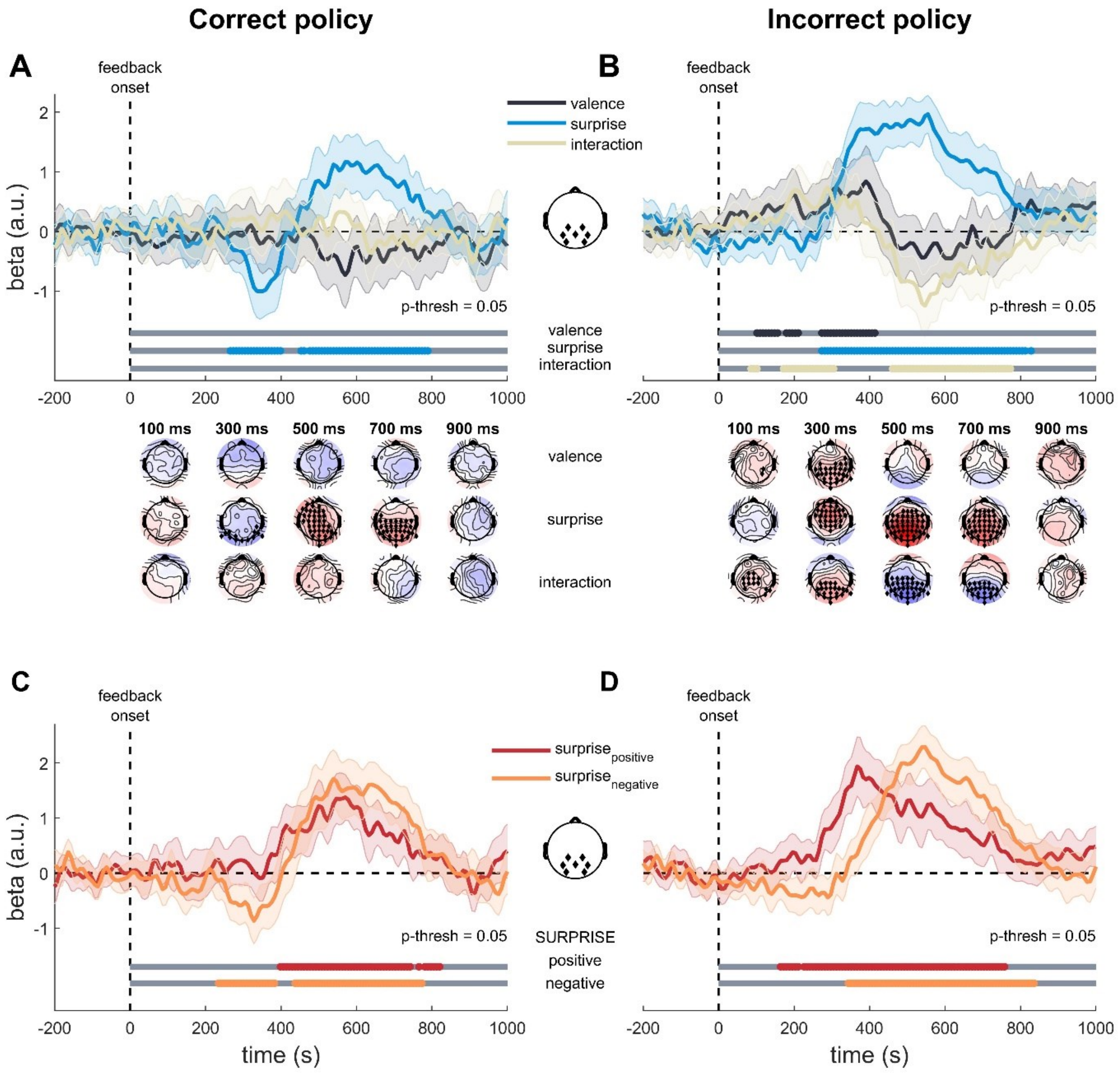

Figure A. Model-based regression analysis for reward prediction errors split into valence and surprise components. A,B. Mean regression (beta) values for valence, surprise and their interaction, separately for the correct and the incorrect policy at posterior electrode sites. C,D. Mean regression (beta) values for the post-hoc regression analysis. Gray bars below indicate the time windows which were considered for cluster-based permutation testing. Colored bars indicate time windows with significant positive and negative effects. Topographies show the significant cluster for the correct policy and the incorrect policy. Black diamonds indicate significant clusters.
