# Supplementary material for: Surprise-minimization as a solution to the structural credit assignment problem
Source: PLoS Comput Biol. 2024 May 28;20(5):e1012175. doi: 10.1371/journal.pcbi.1012175 (PMC11175464; doi:10.1371/journal.pcbi.1012175)
Supplement: Attachment — Submitted filename: Review_PCOMPBIOL-D-23-01284.pdf [file pcbi.1012175.s002.pdf]

**Title:** Surprise-minimization as a solution to the structural credit assignment problem

**Review comments:**

This study by Wurm et al. provides an interesting outlook into how the brain might solve credit assignment problems, utilizing reinforcement learning (RL) models with concurrent learning of multiple policies and model-based analysis of EEG signals. Importantly, the authors have applied the idea of a reliability-based arbitration mechanism that has been previously studied (see also O'Doherty et al., 2021, *Neuroscience & Behavioral Reviews* for a nice review) in the novel context of credit assignment problem. Specifically, the authors employ a novel multi-bandit task design with two distinct choices/outcomes which also work to disambiguate signals for separate "surprise" signals, and present neural correlates in EEG signals during feedback outcome period.

Overall, all components of this study – unique task design, behavioral analysis, model implementation, and EEG analysis – are sound and provide a coherent picture to the question it is addressing. Therefore I would recommend this article for publication, given that the authors address below concerns.

**Major comments:**

*1. Logic for using Gaussian random walks and defining win/loss from graded outcome:*

I understand the Gaussian random walks enable a relatively continuous sampling of stimuli values, but looking at the example trajectory in Figure 1B, there also seems to be large jumps in values that turn a higher-reward stimulus into a lower-reward one in just a single trial. Given such fluctuations, I wonder how the authors' definition of Win-Stay/Lose-Switch strategy remains applicable for this particular task. For example, for the outcome values around 50, the participants might have employed a more random stay/switch strategy given their experience that the reward magnitude could flip in either direction. As such, it is not immediately clear whether they perceive the outcome as a binary "win" or "loss" based on the boundary of 50 points. Regarding this point, have the authors tried a different boundary for win/loss, for example, Win > 60 and Lose < 40? I'm curious if we can expect to see a more clear dissociation between relevant/irrelevant outcomes by using a more conservative value for win/loss.

Also to account for the potential issue for the random walk process, I wonder if it would be possible to quantify how many trials a "correct" stimulus was better-rewarding than the other on average — i.e., how stable the value preferences were for each set of stimuli. This is just to make sure whether Win-Stay/Lose-Switch was a viable strategy to use for the task environment.

*2. Alternative model considerations:*

The two of the control models, *correct* and *incorrect* policy models, rather correspond to very extreme (and unlikely) scenarios where participants exclusively follow either the correct (w=1) or

incorrect (w=0) policy. I think this assumption could be relaxed a little bit by still allowing a minimal mixture for both cases, e.g., w=0.9 for the former and w=0.1 for the latter.

For the full model, I'm also curious whether the authors have tried fitting the initial starting point for arbitration weight as a free parameter itself (instead of initializing at 0.5). In my experience, I found that fitting the initial bias in such arbitration models significantly improves the fit as it captures the potential starting bias in each block. This could be especially beneficial for this task, because participants might put more attention on one arbitrary policy and start adjusting thereafter.

Relatedly, Figure 3D could be made a little more informative – could you also try plotting a time course of abs($\delta$Correct), abs($\delta$Incorrect), and $\Delta$abs($\delta$) as a function of trials, just like Figure3E? This would help the readers see how the 'surprise' is actually minimized over the trials.

*3. Additional EEG analysis*

If I'm not mistaken, the current regression analysis for EEG signals does not distinguish the trial point in the block (e.g., whether it's early or later during the block). I wonder if the authors could perform supplementary analysis that distinguishes them by, for example, comparing the EEG signals during the first 10-15 trials and the last 10-15 trials of the block. If the hypothesis — that the brain is encoding the difference in the surprise signal, $\Delta$abs($\delta$) — is valid, one might expect to see that the difference might not matter so much after the participant has identified the correct policy toward the end of the block. This could provide another window to probe mechanism of how $\Delta$abs($\delta$) is integrated by the brain.

**Minor comments:**

1. I think it might be worth adding a graphical representation of the Transfer task (as Figure 1 E), since this task was paired with the actual bandit tasks and is mentioned throughout the text. This could help readers gain a more intuitive understanding of what it's measuring.
2. #147: It is mentioned that the general structure of the task was instructed to the participants. How explicitly were they instructed about the reward outcomes? E.g., were they just told that the values randomly fluctuate, or were they also informed something about the continuity in the values due to Gaussian random walks? As task instructions are known to significantly influence task performances, the Methods section could clarify this point about the instructions that participants were given.
3. How was a "correct" choice defined for the bandit tasks to measure performance (as used in Figure 2C-D and #294)? For example, is it considered correct whenever the participant chooses the stimulus of value >50 (i.e., higher-reward choice)? If so, I'm not sure if it's appropriate to call it a "correct choice", because the values of the stimuli randomly fluctuated by random walk process, hence being no inherently correct choice options.

4. Relatedly, the Methods section could specify how the starting point of stimuli values were initialized: drawn uniformly from [1, 99] or using certain priors.
5. Are the Pearson correlations reported in #191 and #193 referring to Experiment 1 only, or both tasks combined? (To avoid ambiguity, you could write r and p values within each of the scatter plots.)
6. I assume that the logic for including two variants of the experiment (Experiment 1 and 2) were to dissociate the EEG signals for the two outcomes. Methods section could also make this point explicit by include a sentence or two about the rationale, in #566 (or #593).
7. There seems to be a little conflation for the term "arbitration weight" in the computational model. In #777, I assume it's the $\omega$ value that is initialized as 0, since in Figure 3E, the arbitration should be initialized at 0.5. Perhaps it's more clear to introduce another symbol for $\text{logit}^{-1}(\omega)$ to differentiate when the text is referring to $\omega$ or $\text{logit}^{-1}(\omega)$ as the arbitration weight. I think #264-266 uses $w_{corrmap} = \text{logit}^{-1}(\omega)$.
8. EEG analysis in Figure 5: the figure caption and the word "posterior cluster" in all panels seem to imply that the regression values are only from the "posterior electrode sites." Was this indeed the case, i.e., are the beta values in the panels A-B sampled from posterior site only? I was a little confused because the "significant clusters" in the topographies (indicated with black diamonds) also seem to include non-posterior regions as well. Authors could clarify this point in the figure caption or the main text.
9. #776: Is the decay rate for the unchosen action actually a complement of the learning rate for the chosen action? What's the assumption behind this complementary weighting? Because these are usually considered as two separate free parameters.

**Other minor edits/typos:**

1. Please consider adding equation numbers for all the equations, as it's much easier to refer to them and is considered a standard practice for computational works.
2. Inconsistency in statistics format: in some places for reporting correlation coefficients *r*, zeros before decimal point are dropped following APA format (e.g., #191), whereas other places do not seem to do so. Please make sure to have a consistent formatting choice.
3. #220: "3 blocks á 100 trials": not sure if *á* here was a typo or not, but could be changed to standard English expression to make the meaning more clear. I assume this means 100 trials per each block?
4. Figure 5 caption: Right before the label C (third line of the paragraph), there is a repeated punctuation mark.
