# Supplementary material for: Surprise-minimization as a solution to the structural credit assignment problem
Source: PLoS Comput Biol. 2024 May 28;20(5):e1012175. doi: 10.1371/journal.pcbi.1012175 (PMC11175464; doi:10.1371/journal.pcbi.1012175)
Supplement: Attachment — Submitted filename: PCOMPBIOL_rebuttal_v4.docx [file pcbi.1012175.s003.docx]

**Subject:** Resubmission of Manuscript (PCOMPBIOL-D-23-01284)

Dear Dr. Serre,
dear Dr. Yang,

Thank you for reviewing our submitted paper entitled “Surprise-minimization as a solution to the structural credit assignment problem”. Moreover, we would also like to thank the reviewers for their insightful criticism and suggestions and the overall positive assessment of the scientific premise of our work. We highly appreciate your combined efforts to improve the quality of your manuscript and we hope that the revision meets your expectations.

The new manuscript now contains

- a revised computational model for surprise minimization: Based on the suggestion from Reviewer 1, we ran model selection for variants with free learning decay parameters as well as counterfactual updating. The variants with counterfactual updating clearly outperformed alternative models.
- an additional analysis to predict behavior based on model-derived variables: Based on the suggestion from Reviewer 2, we predicted future behavior based on current action value estimates. In line with the idea of the surprise minimization and arbitration process, we found that accumulated evidence (i.e., arbitration weights) but not trial-to-trial evidence predicts the impact of action values on choice behavior. As expected, the analysis shows how arbitration weights drive action selection towards the more plausible decision-outcome mapping.
- a revised EEG analysis: During the revision process we found a minor mistake in the coding of the surprise signal. It became obvious that the unsigned prediction error signals (as derived from the absolute prediction errors) are indistinguishable from the evidence signal (derived the difference between surprise signals). Therefore, we decided to implement a new regression model with signed prediction errors, evidence signals and arbitration weights. Crucially, we investigated the EEG activity at both the feedback as well as prior to response onset. This analysis further revealed the intricate patterns of credit assignment on the neural level.
- an updated discussion: Based on the suggestions from both reviewers, we implemented recent and relevant research to further highlight the importance of surprise minimization and action arbitration.

You find attached to this letter the responses to the editor’s and reviewers’ comments, with a description of the changes we made. These changes in the manuscript are highlighted in red font color to ease reading. Due to a formatting problem, some changes had to be highlighted as highlights in yellow. We are sorry for the inconvenience.

Please note, that we also updated the inferential statistics for the logistic regression of the behaviora data. We now report z-values, instead of t-values for the test of regression weights.

We hope you will find this thoroughly revised manuscript suitable for publication in PLOS Computational Biology.

Sincerely,

Franz Wurm

## **Reviewer 1**

This study by Wurm et al. provides an interesting outlook into how the brain might solve credit assignment problems, utilizing reinforcement learning (RL) models with concurrent learning of multiple policies and model-based analysis of EEG signals. Importantly, the authors have applied the idea of a reliability-based arbitration mechanism that has been previously studied (see also O’Doherty et al., 2021, *Neuroscience & Behavioral Reviews* for a nice review) in the novel context of credit assignment problem. Specifically, the authors employ a novel multi-bandit task design with two distinct choices/outcomes which also work to disambiguate signals for separate “surprise” signals, and present neural correlates in EEG signals during feedback outcome period.

Overall, all components of this study – unique task design, behavioral analysis, model implementation, and EEG analysis – are sound and provide a coherent picture to the question it is addressing. Therefore I would recommend this article for publication, given that the authors address below concerns.

We thank the reviewer for this positive assessment of our study and will address their raised concerns in detail below.

### **Major comments:**

*1. Logic for using Gaussian random walks and defining win/loss from graded outcome:*

I understand the Gaussian random walks enable a relatively continuous sampling of stimuli values, but looking at the example trajectory in Figure 1B, there also seems to be large jumps in values that turn a higher-reward stimulus into a lower-reward one in just a single trial. Given such fluctuations, I wonder how the authors’ definition of Win-Stay/Lose-Switch strategy remains applicable for this particular task. For example, for the outcome values around 50, the participants might have employed a more random stay/switch strategy given their experience that the reward magnitude could flip in either direction. As such, it is not immediately clear whether they perceive the outcome as a binary “win” or “loss” based on the boundary of 50 points. Regarding this point, have the authors tried a different boundary for win/loss, for example, Win > 60 and Lose < 40? I’m curious if we can expect to see a more clear dissociation between relevant/irrelevant outcomes by using a more conservative value for win/loss. Also to account for the potential issue for the random walk process, I wonder if it would be possible to quantify how many trials a “correct” stimulus was better-rewarding than the other on average — i.e., how stable the value preferences were for each set of stimuli. This is just to make sure whether Win-Stay/Lose-Switch was a viable strategy to use for the task environment.

The reviewer mentions an important issue about the model-free analysis of the behavioral data. In line with previous research (e.g., Findling et al., 2019), we chose the natural inflection point between 1 and 99 as demarcation for wins and losses.

Following the reviewer’s suggestions, we recalculated the regression and planned contrasts with the 60/40 limits for wins/losses. As expected, the results do not change qualitatively: We still find significant effects for relevant outcomes (*z* = 9.42, *p* < .001), irrelevant outcomes (*z* = 4.97, *p* < .001) and their interaction (*z* = 6.94, *p* < .001), and the planned contrast when the relevant outcome alone signaled a win compared to when the irrelevant outcome alone signaled a win (*z* = 4.97, *p* < .001). Please note, that this procedure leads to the exclusion of 16% of the data on average. Even with more conservative thresholds (e.g., 80-20), the results remain stable.

Complementary, we can also plot the psychometric curve to further inspect the relationship between outcome value and stay probabilities (see below). For the relevant outcomes we find a strong sigmoidal curve, indicating win-stay/lose-shift behavior. For the irrelevant outcomes, we still find a sigmoidal curve (but less pronounced) especially at extreme outcome values. We did not add this analysis in the revised manuscript, as we assess the added value to be marginal. However, we are happy to add if the reviewer prefers this alternative way of analyzing the behavioral data.

Addressing the final point on the stability of the random walks, we calculated the average rate for crossing the threshold of 50 (i.e., when an action flips from win to loss or vice versa). On average there are 17.6 flips per 100 trials. This means that the winning response for a stimulus remains stable for approximately 5.7 trials. It is difficult to assess the viability of the WSLS strategy in general. Admittedly, we (over)simplify decision-making and learning in this initial exploration of the data before we proceed to apply computational modeling. Nevertheless, our analyses gain relevant first insights into the behavioral patterns of credit assignment.

Psychometric function for the relationship between outcome values and stay probability on the following trial. Relevant mapping refers to the objectively correct link between decisions and feedbacks, whereas irrelevant mapping refers to the objectively incorrect link.

*2. Alternative model considerations:*

The two of the control models, *correct* and *incorrect* policy models, rather correspond to very extreme (and unlikely) scenarios where participants exclusively follow either the correct (w=1) or incorrect (w=0) policy. I think this assumption could be relaxed a little bit by still allowing a minimal mixture for both cases, e.g., w=0.9 for the former and w=0.1 for the latter. For the full model, I’m also curious whether the authors have tried fitting the initial starting point for arbitration weight as a free parameter itself (instead of initializing at 0.5). In my experience, I found that fitting the initial bias in such arbitration models significantly improves the fit as it captures the potential starting bias in each block. This could be especially beneficial for this task, because participants might put more attention on one arbitrary policy and start adjusting thereafter.

We agree with the reviewer that the chosen models for the correct and incorrect policy constitute rather extreme instantiations. While this was exclusively chosen to make the point about the underlying arbitration mechanism, we share the opinion that this is most probably not realistic. Following the reviewer’s suggestion, we have therefore relaxed the assumptions and also fitted models with arbitration weights of 90 (10%) for the correct (incorrect) policy models. As expected by the reviewer, the 90% model outperforms the strict 100% variant for both the correct and incorrect policy model (XP = 100%). However, both models with fixed arbitration of 90% for the correct and incorrect policy do not compete well with the surprise minimization model (XP = 95%).

We have also incorporated a model with a starting bias parameter. For the behavioral and the EEG experiment, this free parameter does not increase the fit and is still outperformed by the standard model without such a starting point bias. Please note that for the model-based EEG analysis, the starting bias does not alter the results, as the evidence signal is generated from the policies and therefore the trajectory of arbitration weight is not impacted by the starting bias.

Due to clarity, we only report the best fitting model with a surprise minimization mechanism in the main paper. We have added model selection with the different variants for updating of the unchosen options and the starting bias in the supplemental information (Table S1).

Relatedly, Figure 3D could be made a little more informative – could you also try plotting a time course of abs(𝛿Correct), abs(𝛿Incorrect), and Δabs(𝛿) as a function of trials, just like Figure3E? This would help the readers see how the ‘surprise’ is actually minimized over the trials.

Following the reviewer’s comment, we have improved the visualization of the dynamics for our surprise minimization model (see Figure 2). It is clearly visible that there are trial-to-trial fluctuations, the averaged signals nicely converge within a few trials and remain stable throughout the rest of the block. This allows successive evidence accumulation in the long-run.

Time course of the arbitration weight, evidence and surprise signal (=absolute prediction error) within one block. Besides a single-trial example, we also plot averaged signals over 1000 simulations.

*3. Additional EEG analysis*

If I’m not mistaken, the current regression analysis for EEG signals does not distinguish the trial point in the block (e.g., whether it’s early or later during the block). I wonder if the authors could perform supplementary analysis that distinguishes them by, for example, comparing the EEG signals during the first 10-15 trials and the last 10-15 trials of the block. If the hypothesis — that the brain is encoding the difference in the surprise signal, Δabs(𝛿) — is valid, one might expect to see that the difference might not matter so much after the participant has identified the correct policy toward the end of the block. This could provide another window to probe mechanism of how Δabs(𝛿) is integrated by the brain.

This is an interesting additional assumption about the postulated evidence signal Δabs(𝛿). Following the reviewer’s comment, we have investigated this idea but did not find a significant difference for the neural correlates of the evidence signal between the start and the end of a block. While it seems reasonable to assume that the evidence signal loses relevance over the course of a block (as subjects figure out the correct mapping), the lack of such an effect advocates for the robustness of this simple yet effective calculation. It seems that the brain is constantly keeping track of the evidence for different world models, allowing it to flexible adapt to changing environments.

However, we have added an additional model-based EEG analysis (line 441-454). This analysis was added as a response to a comment from Reviewer 2. We show that the arbitration weight, reflecting the successive accumulation of evidence, is reflected in the response-locked data.

### **Minor comments:**

1. I think it might be worth adding a graphical representation of the Transfer task (as Figure 1 E), since this task was paired with the actual bandit tasks and is mentioned throughout the text. This could help readers gain a more intuitive understanding of what it’s measuring.

We agree with the reviewer and have added a graphical representation of the transfer task in Figure 1, alongside with a brief description in the figure caption.

2. #147: It is mentioned that the general structure of the task was instructed to the participants. How explicitly were they instructed about the reward outcomes? E.g., were they just told that the values randomly fluctuate, or were they also informed something about the continuity in the values due to Gaussian random walks? As task instructions are known to significantly influence task performances, the Methods section could clarify this point about the instructions that participants were given.

Participants were instructed and trained on the anti-correlated and continuous nature of the walking bandit. Anti-correlation was introduced by the statement that within one decision there are always exactly 100 coins distributed between the stimuli. Continuity was introduced by the statement that on each trial only a fraction of the coins is re-assigned. Participants were also provided with a tabular example of such an anti-correlated and continuous random walk. In this example, there was a flip in “win” and “loss” (values crossing the inversion point of 50).

As suggested by the reviewer, we have further clarified the instruction to participants in the methods section (line 679-686).

3. How was a “correct” choice defined for the bandit tasks to measure performance (as used in Figure 2C-D and #294)? For example, is it considered correct whenever the participant chooses the stimulus of value >50 (i.e., higher-reward choice)? If so, I’m not sure if it’s appropriate to call it a “correct choice”, because the values of the stimuli randomly fluctuated by random walk process, hence being no inherently correct choice options.

We agree that the term “correct” is misleading in this context. In the revised manuscript we therefore refrain from using this term and have replaced “correct choices” with “wins”, as wins are clearly defined as choices that lead to outcomes with a value larger than 50.

4. Relatedly, the Methods section could specify how the starting point of stimuli values were initialized: drawn uniformly from [1, 99] or using certain priors.

The starting values were drawn uniformly from the interval [1, 99]. For clarity, we have added this information in the methods section (line 633f).

5. Are the Pearson correlations reported in #191 and #193 referring to Experiment 1 only, or both tasks combined? (To avoid ambiguity, you could write r and p values within each of the scatter plots.)

The reviewer is correct. The correlation values are only referring to Experiment 1. The respective values for Experiment 2 are only introduced later (line 374f). We have added the r and p values in the figure.

6. I assume that the logic for including two variants of the experiment (Experiment 1 and 2) were to dissociate the EEG signals for the two outcomes. Methods section could also make this point explicit by include a sentence or two about the rationale, in #566 (or #593).

The rationale for the different variants of the experiment was exactly as described by the reviewer. We have made the distinction between Experiment 1 (behavioral) and Experiment 2 (EEG) clearer in the methods section (line 637f).

7. There seems to be a little conflation for the term “arbitration weight” in the computational model. In #777, I assume it’s the 𝜔 value that is initialized as 0, since in Figure 3E, the arbitration should be initialized at 0.5. Perhaps it’s more clear to introduce another symbol for logit-1(𝜔) to differentiate when the text is referring to 𝜔 or logit-1(𝜔) as the arbitration weight. I think #264-266 uses wcorrmap = logit-1(𝜔).

We appreciate the reviewer’s eye for detail and have adjusted the respective parts in the manuscript. Moreover, we have clarified that the arbitration weight is initialized as 0 (i.e., 0.5 after the inverse logit transformation) (line 837ff).

8. EEG analysis in Figure 5: the figure caption and the word “posterior cluster” in all panels seem to imply that the regression values are only from the “posterior electrode sites.” Was this indeed the case, i.e., are the beta values in the panels A-B sampled from posterior site only? I was a little confused because the “significant clusters” in the topographies (indicated with black diamonds) also seem to include non-posterior regions as well. Authors could clarify this point in the figure caption or the main text.

We are thankful that the reviewer has spotted this inaccuracy in the caption of figure 5. It is true that the cluster contains electrodes beyond only posterior electrodes. However, as visible from the topographies, the cluster is concentrated at posterior electrode sites. This is also a common location for feedback-locked ERPs, which is why we plotted only parieto-central electrodes. We refrain from using the term in the figure, have clarified the channels that constitute the “posterior cluster” in the caption and added an additional panel in the figure.

9. #776: Is the decay rate for the unchosen action actually a complement of the learning rate for the chosen action? What’s the assumption behind this complementary weighting? Because these are usually considered as two separate free parameters.

Our choice for setting the decay rate for unchosen options as the complement of the learning rate was based on previous research (Barraclough et al., 2004). Following up on the reviewer’s comment we have investigated the idea of an additional free parameter for learning decay of the unchosen options. In addition to a model with an independent learning decay parameter, we have also added a model with counterfactual updating. Such a counterfactual updating mechanisms is sensible given the anti-correlated nature of the random walk procedure for each bandit. This anti-correlation was also transparent from the instruction text.

Interestingly, model comparison clearly points towards the counterfactual model when compared directly to the complementary decay model and the independent decay model. Given that the counterfactual model outperforms the other model variants, we have updated the manuscript. This led to a few minor changes in the behavioral and EEG result but did not change the main results that the lower-level prediction errors and higher-level evidence signals are reflected in the feedback-locked EEG signal. Please note, that we chose to discuss model comparison for additional variants with the surprise minimization in the supplemental information (see also model with starting bias).

**Other minor edits/typos:**

1. Please consider adding equation numbers for all the equations, as it’s much easier to refer to them and is considered a standard practice for computational works.

The revised manuscript now contains equation numbers for all equations.

2. Inconsistency in statistics format: in some places for reporting correlation coefficients *r*, zeros before decimal point are dropped following APA format (e.g., #191), whereas other places do not seem to do so. Please make sure to have a consistent formatting choice.

Correlation coefficients are now consistently reported in the revised manuscript.

3. #220: “3 blocks á 100 trials”: not sure if *á* here was a typo or not, but could be changed to standard English expression to make the meaning more clear. I assume this means 100 trials per each block?

The reviewer is correct. There were 100 trials per block and 3 blocks in total. We have clarified the issue by using the reviewer’s suggested expression (line 230).

4. Figure 5 caption: Right before the label C (third line of the paragraph), there is a repeated punctuation mark.

We have deleted the repeated punctuation mark.

## **Reviewer 2**

Wurm et al. studied neurocomputational mechanism of discovering decision-outcome mapping when it is hidden and needs to be discovered. For this purpose, the authors designed a task in which participants made two separate two-armed bandit choices and received separate outcomes, while the link between each decision and outcome was unknown and had to be discovered. The authors showed that participants learnt the task. They then designed a computational model based on competing policies: two different policies competed for explaining the behaviour. The policy that led to the smaller surprised had more influence on behaviour. The influence of each policy was determined by comparing the surprise of each policy. Using EEG, the authors indicated that neural activity was correlated with surprise obtained from the two competing policies (though it was greater for the correct policy). They also showed neural correlates of the difference between the surprise signals obtained from the two policies.

The study is interesting, and the research question is very timely. The manuscript is in general well-written, however I had trouble working out some behavioural analyses because of the lack of clarity in the way that the analyses were conducted and in the representation of the data. More importantly, I believe the authors do not provide adequate evidence for the “surprise minimisation” account which they try to advance. In addition, some of the statistical procedure seem incorrect to me. Please see my comments below.

We are grateful for the comments of the reviewer and hope that we could clarify the central issues in our revised manuscript and thereby strengthened our argument for the surprise minimization account for solving the credit assignment problem.

1) In their introduction and discussion and the title of the paper, the authors suggest that surprise-minimisation is a solution and mechanism for solving the task. Their evidence for this account is the neural correlates of surprise that the study showed using EEG. I understand why the authors think “surprise minimisation” can be used to explain their results, because of the free energy principle. But if the authors wish to do so, they need to provide evidence that “surprise minimisation” is what the brain and the participants were doing for solving this task. The authors reviewed some articles related to the free energy principle. The free energy principle is not falsifiable (suggested by others and recently by its advocates too, please see <https://www.sciencedirect.com/science/article/pii/S037015732300203X>). So, my question is, how does this study provide evidence for the statement that the surprise minimisation is the solution for solving this task?

We thank the reviewer for this clarifying question. Most importantly, the evidence for our surprise minimization account stems from the model comparison: we show that a model with surprise minimization explains the data better than alternative models with a different or even without such a mechanism. We have included a clarification in the manuscript (line 465f). Additionally, we provide evidence for our account by simulation: The model infers the correct mapping between decisions and feedback color in more than 99% of the cases. Finally, we provide evidence for the biological plausibility of this solution by showing that the central model variable –the evidence signal that pushes the arbitration – is reflected in the neural signals of human participants. Moreover, we refer to surprise minimization as merely “a” (of possibly many) but not “the” (one and only) solution for solving the task.

Please note, that we neither interpret the current findings as evidence for the free energy principle, nor advocate the free energy principle as such. We point out a similarity between both ideas: the central importance of prediction errors for solving a causal inference problem. Following the reviewer’s suggestions below, the revised manuscript now highlights the computational role of surprise besides the similarity with the free energy principle and other current frameworks (line 475-500).

I recommend the authors to focus on the computational role of surprise to solve the task. Obviously, it is up to the authors to speculate about the consistency of their results with free energy principle, but it should not prevent them from presenting a more detailed computational account of their data. Critically, the surprise computed for each model can be used as the evidence in favour of each policy (as the authors compute). This is not the first study that investigates the effect of surprise in learning and decision making. Some other studies have investigated how surprise can be used to guide behaviour. For example, Harsmaa et al. Moceluar Psychiatry 2020 have studied the effect of surprise. In addition, Mahmoodi et al. BioRxiv 2023 have studied latent cause inference using surprise as a measure of solving the latent cause inference task. I think the authors should take a similar approach and discuss the relevant literature like those mentioned above. However, if they want to insist on “surprise minimisation” as it is suggested by the free energy principle, they have to provide evidence for their account, a feat which I’m afraid has been evading the advocates of the theory and eventually led to concede it is not a falsifiable theory.

We fully agree with the reviewer’s suggestion. As outlined above, we do not advocate for the free energy principle, but simply aimed to highlight the relevance of surprise signals and their use for higher-level inference. The revised manuscript now contains an extended discussion on the computational role of surprise as well as reference to the papers cited by the reviewer (line 487f and line 558ff). We thank the reviewer for pointing us towards this highly relevant and current literature.

By investigating the computational role of surprise in the task, the authors can ask more detailed and informative questions. For example, the difference in surprise signal (separately for each choice) can be used on the next trial as the “evidence signal”-- the probability that one policy is better than the other -- to guide the choice. The authors should run this analysis. The existence of such a signal indicates that the surprise signal is used to compute the likelihood that each policy is right. It will offer a computational role for surprise, similar to recent studies (e.g., Mahmoodi et al. BioRxiv 2023).

We thank that reviewer for this insightful suggestion for an additional analysis. We ran and incorporated the respective analysis that aimed to predict upcoming choice behavior based on the difference between the estimated action values (line 296-313 for Experiment 1 and line 377-386 for Experiment 2). In line with our expectations, we show that action values for the different policies predict upcoming choices. Crucially, this pattern is not modulated by the trial-to-trial evidence but the accumulated evidence as reflected in the arbitration weight. While the impact of action values from the correct policy increases with accumulated evidence, the impact from the incorrect policy decreased. We think this additional analysis offers valuable insights into the computational role of surprise and behavioral arbitration.

Based on this insightful comment, we have also added a new model-based EEG analysis, where we predict response-locked data (line 441-454). Crucially, we show that the arbitration weights are reflected in the EEG prior to response onset. We see this as clear evidence that action selection is driven by a process akin to our postulated arbitration mechanism.

2) I found it very difficult to work out what Figure 2A-B and 3A are supposed to communicate. In figure 2, we have win/loss on x-axis, and we also have grey/white colours, but is not clear what each colour is. In addition, x-axis label indicates it is relevant outcome. moreover, on panel 2B there is a label at the top right which reads (win/loss for grey and white colours). How’s this labelling different from win/loss on x-axis?

We thank the reviewer for pointing out an ambiguity in our visualization. We have added more information in the text (line 176ff), the relevant figures and their captions.

3) As far as I understood, there is an effect of irrelevant win on stay probability. Is it not a failure of credit assignment? It indicates that if we you receive a good outcome, even it is irrelevant, you are more likely to repeat your current policy. This is a confusion effect. How do the authors explain it?

The reviewer interprets the main effect of the irrelevant outcome as a failure of credit assignment or a confusion effect. We explain this effect based on our arbitration mechanism: Arbitration starts in a state of uncertainty and slowly converges to the correct representation. This means that irrelevant outcomes always exert influence on action selection (as shown in the reviewer’s suggested analysis). Accumulated evidence never reaches “perfect” arbitration. Therefore, we interpret this effect as a inherent consequence of the arbitration mechanism itself (line 468ff).

4) There is a study which indicates that the permutation test cannot be used to make any statement about the onset and offset of an effect (<https://onlinelibrary.wiley.com/doi/10.1111/psyp.13335>). I strongly urge the authors to report whether their procedure is consistent with this study. I am baffled by the significance of the red curve in Figure 5B, right panel. It does not seem significant to me even without applying any correction. The significance line indicates significance even for areas which the error bar almost overlaps with the zero line. How is that possible? I think the authors made the unwarranted interpretations of the permutation test that the study that I referred to above has addressed. If this is the case, the authors should correct their procedure.

We fully agree with the reviewer on this matter and we were aware of the inferential issues for the interpretation of the results from permutations tests, as promoted in the above stated paper by Sassenhagen and Draschkow (2019). Following their recommendations, the original manuscript did not make any inferential claims about absolute temporal onsets and offsets or relative dynamics between the different regressors. However, we realized that our reporting of the results is not in line with the best practice as promoted by Sassenhagen and Draschkow (2019). The revised manuscript now reports the extent of clusters explicitly more descriptive and also explicitly mentioned the paper in the methods section (line 946ff). Importantly, the main conclusion of our study (model-based evidence signal is reflected in the neural data) is still a valid interpretation from the cluster-based permutation test.

Please note, regarding the surprising significance and discrepancy between the curve in Figure 5B, we want to clarify that the curve is the average beta for all involved electrodes. In contrast, the significance of the cluster signifies if at least one electrode in the involved electrodes participated in the cluster at that time point. This minor discrepancy between the significance and the curves can explain the otherwise surprising conflict in the data.

## **References**

Barraclough, D. J., Conroy, M. L., & Lee, D. (2004). Prefrontal cortex and decision making in a mixed-strategy game. *Nature Neuroscience*, *7*(4), 404–410. https://doi.org/10.1038/nn1209

Findling, C., Skvortsova, V., Dromnelle, R., Palminteri, S., & Wyart, V. (2019). Computational noise in reward-guided learning drives behavioral variability in volatile environments. *Nature Neuroscience*, *22*(12), 2066–2077. https://doi.org/10.1038/s41593-019-0518-9

Ito, M., & Doya, K. (2009). Validation of decision-making models and analysis of decision variables in the rat basal ganglia. *Journal of Neuroscience*, *29*(31), 9861–9874. https://doi.org/10.1523/JNEUROSCI.6157-08.2009
