# Supplementary material for: Surprise-minimization as a solution to the structural credit assignment problem
Source: PLoS Comput Biol. 2024 May 28;20(5):e1012175. doi: 10.1371/journal.pcbi.1012175 (PMC11175464; doi:10.1371/journal.pcbi.1012175)
Supplement: Attachment — Submitted filename: RebuttalLetter_PLOSCB_round2_v2.docx [file pcbi.1012175.s004.docx]

**Subject:** Resubmission of Manuscript (PCOMPBIOL-D-23-01284)

Dear Dr. Serre,
dear Dr. Yang,

Thank you for reviewing our re-submitted paper entitled “Surprise minimization as a solution to the structural credit assignment problem”. Additionally, we would also like to thank the two reviewers for their second round of insightful feedback and suggestions for improvement. We highly appreciate your combined efforts and the overall positive assessment of the manuscript and its possible publication.

You find attached to this letter the response to the reviewers’ comments with a description of the changes we made on their basis. The changes in the manuscript are highlighted in red font color to ease their detection and processing.

We hope you find the revisions suitable for publications in PLOS Computational Biology.

Sincerely,

Franz Wurm

**Reviewer #1**

All of my comments were addressed adequately, and I appreciate the authors' efforts on re-running the analyses and fitting new models. Overall I think the paper has been strengthened.

We thank the reviewer for this positive feedback on the revisions.

Below are just additional minor comments and follow-ups to the authors' response.

**Comment 1:** Thank you for checking this analysis. I agree that the new figure adds marginal value and better be excluded from the manuscript. Although, I find it very interesting that the psychometric curve for irrelevant mapping (red) seems to have overall stay bias (as the middle point at value of 50 seems to have over chance level of staying). Just wondering if the authors have insight to this effect?

The reviewer raises an interesting question. The general tendency to stay with previous behavior (compared to switch) irrespective of previous outcome, is a common finding in human research with bandit tasks. For example, already early studies of the two-stage Markow decision task report strong stay behavior above 50% even after negative feedback (e.g., Daw et al., 2011). Our own past research supports this patterns (Wurm et al., 2020, 2021).

To account for this behavioral tendency, computational models routinely incorporate a perseveration (or choice hysteresis) parameter. This parameter captures biases in decision making, i.e., overly strong stay or switch behavior. On a meta-analytical level, this parameter is demonstrated to be robust (Palminteri, 2022). Possible mechanistic explanations for choice hysteresis include decreasing learning rates (over time) as well as noisy updating of action values. To capture these pervasive patterns in human behavior, the current manuscript also contains a perseveration parameter (see Eq. 10). In line with a wealth of research, the perseveration parameter was significantly different from zero for both Experiment 1 (*M*=0.33, *SEM*=0.07; *t*(47)=4.86, *p* < .001) and Experiment 2 (*M*=0.33, *SEM*=0.03; *t*(27)=8.01, *p* < .001).

Interestingly, the increased tendency to stay with previous behavior for irrelevant outcomes compared to relevant outcomes is also evident in the simulated data (see figure below). This suggests that this behavioral pattern is a byproduct of correct credit assignment: The incorrect mapping loses importance for action selection and therefore the psychometric curve flattens out. Additionally, random walks for both outcomes are independent. As performance is above chance and driven by the correct policy, switch behavior following irrelevant outcomes approaches average switch behavior for relevant outcomes.

Psychometric function for the relationship between outcome values and stay probability on the following trial for the behavioral data (left panel) compared to simulated data (right panel). Relevant mapping refers to the objectively correct link between decisions and feedbacks, whereas irrelevant mapping refers to the objectively incorrect link. Simulated data are generated following the same procedures as described in the manuscript (1000 runs, empirical priors for parameters).

For the average crossing rate (17.6 flips per 100 trials, stable for 5.7 trials) -- I think this detail is worth reporting and can be mentioned in the Methods for the task details.

We agree with the reviewer. Therefore, the revised manuscript now reports the average stability for the random walk procedure in the methods section (line 641f).

**Comment 2:** Thank you for testing this additional models. I see how the additional starting bias is not helping with the fit, although decreasing the -log-likelihood. Table S1 is clear.
Figure 3E is also nice. A very minor styling preference: totally up to your choice, but since the third panel already says "Surprise" the y-label could be written as |δ|, consistent with the top panel. Y-label for Evidence generation could be Δ as well.
Also in the figure caption, it feels a little awkward that the order of explanation is from bottom to top panels, instead of top to bottom. Also there is a typo: "middle pane."

We thank the reviewer for pointing out this inconsistency in the panel order and axis labels. We have reversed the order of the panels, so that it follows the mechanistic flow from surprise generation to evidence accumulation.

Comment 3: Thank you for testing this hypothesis. The results are interesting on their own, as they suggest that the brain is encoding the signal robustly. The new EEG analysis also shows nice separation between feedback and response periods. I refer additional comments for it to Reviewer 2.

We appreciate the reviewer’s evaluation of our additional EEG analysis.

**Other additional comments:**

- Typo in Eq. 4: dd_i should be d_i?

We thank the reviewer for their eye for detail. We corrected this mistake.

- Just caught this but in Eq. 9, the arbitration weight for the incorrect option should be (1-logit^-1(ω)) instead of logit^-1(1-ω), according to your implementation and the codes. Note that according to current formulation in Eq. 9, the two sigmoids (for correct & incorrect arbitration weights) are symmetric around ω of 0.5, not 0.

The reviewer correct. This revised manuscript now reports the correct equation as 1-logit^-1(ω).

- Relatedly for Eq. 12, shouldn't the decision value for incorrect (D_incorrect) be multiplied with (1-logit^-1(ω)), instead of the current formulation which multiplies arbitration weight for "correct" decision value?

Please note that equation 12 describes a regression model. The asterisk does not indicate a literal multiplication but rather indicates an interaction term between the respective variables. Therefore, the inversion (1-logit) would not change the results but rather change the sign of the resulting beta values. We chose to incorporate the interaction without this inversion in order to avoid unnecessary inflation of regressors.

- Line #927: (Eq. 13) → (Eq. 14)

We have updated the reference to the equation in the text.

**Reviewer #2:**

The authors have addressed most of my previous comments.

We are glad to hear that the revised manuscript addresses most comments appropriately.

However please see my remaining concern below.

I still find Figure 2A-B and 3A-C very confusing. For example, in Figure 2A we have win and loss on the x-axis. On the top right corner of Figure 2B, win and loss are color coded with dark and bright colours. On top of the color code it reads irrelevant outcome. I find these details very confusing. Can you please clarify these panels?

We understand the reviewer’s confusion about Figure 2A-B and 3A-C. It is uncommon that a bandit task affords more than a single win/loss dimension. However, due to the nature of our modified version with two decisions and two outcomes, each decision (stay/switch) is classified under both the correct mapping (i.e., relevant win/loss) and the incorrect mapping (i.e., irrelevant win/loss). To avoid confusion for the reader, we have added a more detailed explanation in the manuscript (line 168ff) and a brief example in the caption of Figure 2.

**References**

Daw, N., Gershman, S. J., Seymour, B., Dayan, P., & Dolan, R. J. (2011). Model-based influences on humans’ choices and striatal prediction errors. *Neuron*, *69*(6), 1204–1215. https://doi.org/10.1016/j.neuron.2011.02.027

Palminteri, S. (2022). Choice-Confirmation Bias and Gradual Perseveration in Human Reinforcement Learning. *Behavioral Neuroscience*, *137*(1), 78–88. https://doi.org/10.1037/bne0000541

Wurm, F., Ernst, B., & Steinhauser, M. (2020). The influence of internal models on feedback-related brain activity. *Cognitive, Affective, & Behavioral Neuroscience*. https://doi.org/10.3758/s13415-020-00820-6

Wurm, F., Walentowska, W., Ernst, B., Severo, M. C., Pourtois, G., & Steinhauser, M. (2021). Task learnability modulates surprise but not valence processing for reinforcement learning in probabilistic choice tasks. *Journal of Cognitive Neuroscience*, *34*(1), 34–53. https://doi.org/10.1162/jocn_a_01777
